# Study of Complete Surgical Response of Stage III Oral Carcinoma Patients in Comparison to Primary Surgery Versus Neoadjuvant Methotrexate With Surgery

**DOI:** 10.7759/cureus.72680

**Published:** 2024-10-30

**Authors:** Siddharth B Lonare, V. Manoj Babu, Rajat Sharma, Krunal A Chendkapure, Abhijeet S Velip, Nirbhay Bind

**Affiliations:** 1 General Surgery, Byramjee Jeejeebhoy Government Medical College and Sassoon General Hospital, Pune, Pune, IND; 2 General Surgery, Maharishi Markandeshwar Medical College and Hospital, Solan, IND; 3 Surgical Oncology, All India Institute of Medical Sciences, Rishikesh, Rishikesh, IND

**Keywords:** chemotherapy alternatives, negative surgical margins, neoadjuvant methotrexate therapy, oncological outcomes, oral cancer surgery, stage iii oral carcinoma, tumor downstaging

## Abstract

Objective

This study aims to compare the efficacy of neoadjuvant methotrexate therapy followed by surgery versus primary surgical management in patients with stage III oral carcinoma.

Methods

Thirty patients diagnosed with stage III oral carcinoma were enrolled in this prospective study at a tertiary cancer research center. The patients were divided into two groups: 15 patients received neoadjuvant methotrexate therapy followed by surgery, while 15 underwent primary surgical management. Outcomes were evaluated based on tumor downstaging, surgical margins, postoperative complications, and the requirement for adjuvant radiotherapy.

Results

Patients in the neoadjuvant methotrexate group demonstrated significant tumor downstaging, allowing for less extensive surgical procedures, with 33.3% (n=5) undergoing wide local excision (WLE) compared to 13.3% (n=2) in the primary surgery group. Negative surgical margins were achieved in 93.33% (n=14) of patients in the neoadjuvant group versus 53.33% (n=8) in the surgical group. Additionally, only 13.3% (n=1) of patients in the neoadjuvant group required postoperative radiotherapy, compared to 53.33% (n=8) in the surgical group. The recurrence rate over a six-month follow-up period was comparable between the two groups.

Discussion

Neoadjuvant methotrexate therapy resulted in better surgical and oncological outcomes by downstaging the tumor, reducing the extent of surgery, and minimizing the need for postoperative radiotherapy. The findings suggest that methotrexate, as a neoadjuvant agent, is effective in improving patient outcomes with fewer side effects compared to standard cisplatin-based regimens.

Conclusion

Neoadjuvant methotrexate therapy offers a viable treatment option for stage III oral carcinoma, demonstrating improved oncological and surgical outcomes, including less invasive surgery, higher rates of negative surgical margins, and reduced postoperative radiotherapy requirements. Further research with long-term follow-up is necessary to validate these findings and explore the long-term impact on survival and recurrence rates.

## Introduction

Oral carcinoma is one of the most prevalent cancers in India, with an incidence rate two to three times higher in men than in women [1]. According to GLOBOCAN 2008, oral cancers affect nearly a quarter of a million individuals each year, making it the most common head and neck cancer worldwide [2]. Oral cancer primarily involves the structures within the oral cavity, which include the lips, inner cheeks (buccal mucosa), gums (gingiva), the front two-thirds of the tongue, the floor of the mouth, the hard palate, and the retromolar trigone [3], with more than 90% being squamous cell carcinomas. These carcinomas are predominantly linked to exogenous factors such as tobacco smoking [4] and according to the National Institute on Alcohol Abuse and Alcoholism (NIAAA), Excessive alcohol consumption is defined as over 14 drinks per week for men and over seven drinks per week for women [5].

As per GLOBOCAN 2008, there were around 260,000 new cases of oral cavity cancer with a mortality of 83,000 patients, worldwide [2]. Smoking is a major risk factor for oral carcinoma, with tobacco use contributing to the majority of cases worldwide and up to 75% of oral cancers in certain populations [6]. In India, low-income groups are disproportionately affected due to the high prevalence of tobacco chewing and limited access to advanced diagnostic tools, often leading to delayed diagnosis of oral cancer [7]. Management of oral carcinoma is largely dependent on the stage at diagnosis. For stage III oral carcinoma, standard treatment typically involves wide local excision (WLE) of the tumor combined with supra-omohyoid neck dissection, targeting lymphatic drainage areas including level IA (submental triangle), IB (submandibular triangle), and II (upper deep jugular nodes). Methotrexate, a dihydrofolate reductase (DHFR) inhibitor, is used as a neoadjuvant therapy in this study. By competitively inhibiting DHFR, methotrexate interferes with tetrahydrofolate synthesis, which is crucial for DNA replication [8]. Methotrexate was chosen as neoadjuvant therapy in this study due to both clinical considerations and patient-specific factors. Although cisplatin-based regimens are widely recommended as the standard according to guidelines such as those from the NCCN [9], methotrexate has an established role in the treatment of squamous cell carcinoma, especially in head and neck cancers [10]. For patients who cannot tolerate more aggressive treatments like cisplatin-whether due to factors such as advanced age, pre-existing comorbidities, or compromised renal function-methotrexate provides a viable alternative with fewer side effects [11]. In practice, especially in resource-limited settings, methotrexate is a practical and cost-effective option, offering a balance between effective tumor reduction and maintaining patient safety [12]. This treatment approach ensures that patients receive effective care while minimizing the risk of complications, in line with evidence-based practices that focus on individualized treatment strategies [13].

This study aims to evaluate the effectiveness of methotrexate as a neoadjuvant therapy in facilitating surgical resection by downstaging tumors in patients with stage III oral carcinoma. In this study, we examine patients with stage III oral carcinoma, comparing outcomes between those who undergo surgery alone and those who receive methotrexate as neoadjuvant therapy before surgery. The primary outcomes assessed are early negative margins and recurrence rates.

According to Ferlay et al., approximately 300,000 cases of oral cancer were reported globally in 2012 [14], accounting for 2.1% of all cancers, with around 145,000 fatalities. The Surveillance, Epidemiology, and End Results (SEER) program reports a five-year survival rate of 62.2% for oral carcinoma, with a significantly lower survival rate of 20% for late-stage diagnoses, compared to 82% for early-stage (localized) tumors [15].

## Materials and methods

Our study was a single-centered, prospective, randomized controlled trial conducted at the Department of General Surgery, Byramjee Jeejeebhoy Government Medical College and Sassoon General Hospital, Pune Government Medical College and Sassoon General Hospital, Pune (IEC No. 1218226-226). The study took place over a duration from December 21, 2018 to December 1, 2020. Ethical approval was obtained from the institutional ethical committee. All patients aged 18 years and older with a diagnosis of primary stage III oral carcinoma were included in this study. Stage III oral carcinoma was chosen because it represents a critical stage where the tumor is locally advanced but still amenable to resection, with a significant risk of regional lymph node involvement and recurrence. Patients with stage I, II, or IV oral carcinoma, tumors involving hard palate, soft palate, or retromolar trigone patients with advanced co-morbidities like uncontrolled diabetes mellitus, hypertension, seropositive status like HIV, Hepatitis B or Hepatitis C, and patients unwilling to take part were excluded in this study. 

Following the inclusion and exclusion criteria, 30 patients were included in this study and were randomized into two groups, sample randomization was performed using a computer-generated random number list. The first group called the neoadjuvant group, involved patients undergoing neoadjuvant chemotherapy with methotrexate followed by surgery. The neoadjuvant regime used for all patients in this group was standardized at two doses of methotrexate (45 mg/m²) [16] on days 1 and 14, followed by surgery on day 30. The second group called the surgical group, had primary WLE. 

The treatment protocol for the neoadjuvant group was as follows: Methotrexate doses were calculated based on body surface area (average of 1.9 m² for men and 1.6 m² for women), resulting in a dose of 85.5 mg for men and 72 mg for women [16]. Patients in both groups underwent surgical resection tailored to the location of the tumor, followed by radiotherapy as indicated following MDT discussion. 

These patients were then followed up in the clinic after six months with diagnostic investigations and follow-up tests. These tests were standard for all 30 patients and included the following - routine blood investigations (full blood count (FBC), C-reactive protein (CRP), liver function test (LFT), renal function test (RFT)) were performed on postoperative day 1, x-ray of chest and mandible on postoperative day 7. CT scans of the head, neck, and face on postoperative day 30, PET-CT scans to assess recurrence/metastases at three months, and Biopsies for histopathological evaluation [17].

The data collected included demographic data, clinical staging, surgical parameters, pathological staging, and lymph node involvement. These were then analyzed with statistical tests using SPSS software (IBM Corp., Armonk, NY). A p-value < 0.05 is considered statistically significant.

## Results

The comparison of age and gender distribution between the neoadjuvant methotrexate group and the surgical management group revealed no statistically significant differences. The mean age in the neoadjuvant methotrexate group was 49 years, compared to 46.73 years in the surgical management group, with a p-value of 0.1468, indicating that both groups were comparable in terms of age distribution. Similarly, the gender distribution showed that 80% of the patients in the neoadjuvant group were male, compared to 86.67% in the surgical group, while female patients comprised 20% and 13.33% of the respective groups. The p-value of 0.628 confirms that the difference in gender distribution between the two groups was not statistically significant (Table 1).

**Table 1:** Comparison of age and gender characteristics between neoadjuvant methotrexate and surgical management groups Age (years) - Mean, SD: P-value calculated using an unpaired t-test. Gender (Male, Female):  P-value calculated using a Chi-square test.

Characteristic	Neoadjuvant methotrexate group (N=15)	Surgical management group (N=15)	P-value
Age (years) - Mean	49	46.73	0.1468
Age (years) - SD	7.86	9	
Gender - Male	12	13	0.628
Gender - Female	3	2	

At diagnosis, the TNM staging distribution showed that most patients in the neoadjuvant methotrexate group presented with advanced local disease, characterized by regional lymph node involvement but no distant metastasis. Specifically, 6.67% of patients were staged as T1N1M0, while the majority were classified as T2N1M0 and T3N1M0, each accounting for 46.67% of the patients. This distribution highlights that a significant proportion of patients presented with more advanced stages of the disease (Table 2).

**Table 2:** TNM staging before neoadjuvant methotrexate therapy

	TNM staging	Frequency (before neoadjuvant therapy)	Proportion % (before neoadjuvant therapy)
0	T1N1M0	1	6.67
1	T2N1M0	7	46.67
2	T3N1M0	7	46.67

Following neoadjuvant methotrexate therapy, there was a marked downstaging effect. The percentage of patients with T2N1M0 decreased from 46.67% to 33.3%, and those with T3N1M0 decreased from 46.67% to 20%. These findings suggest that neoadjuvant therapy played a significant role in reducing the tumor burden and downstaging the disease, improving the patients' surgical and oncological outlook (Table 3).

**Table 3:** TNM staging after neoadjuvant methotrexate therapy

	TNM staging	Frequency (after neoadjuvant therapy)	Proportion % (after neoadjuvant therapy)
1	T1N0M0	2	13.3
2	T1N1M0	2	13.3
3	T2N0M0	2	13.3
4	T2N1M0	5	33.3
5	T3N0M0	1	6.7
6	T3N1M0	3	20

There was a statistically significant reduction in the incidence of lymph node malignancy in patients who underwent neoadjuvant methotrexate therapy compared to those who received only surgical management. In the neoadjuvant group, 26.7% of patients showed lymph node malignancy in HPE, compared to 66.7% in the surgical management group. Conversely, 73.3% of patients in the neoadjuvant group had no lymph node malignancy, compared to 33.3% in the surgical group. The p-value of 0.03 indicates that this reduction is statistically significant (Table 4).

**Table 4:** Incidence of lymph node malignancy in HPE P-value was calculated using the Chi-square test

Lymph node malignancy in HPE	Neoadjuvant methotrexate group (N=15)	Surgical management group (N=15)	P-value
Yes	4 (26.7%)	10 (66.7%)	0.03
No	11 (73.3%)	5 (33.3%)	

The most common site of the lesion in both groups was the gingivobuccal region, accounting for 40% of cases in the neoadjuvant methotrexate group and 60% in the surgical management group [18]. Both groups had a similar distribution of lesions on the lower lip, with 26.7% of patients in each group. The anterior half of the tongue was involved in 13.3% of the neoadjuvant group and 6.7% of the surgical group. Other less common sites included the junction of the right oral commissure of the lips and the lower gingival region in the neoadjuvant group, while the surgical group had lesions on the right side of the tongue (Table 5).

**Table 5:** Distribution of site of lesion between study groups

Site of lesion	Neoadjuvant methotrexate group (N=15)	Surgical management group (N=15)
Junction of right oral commissure of lips	1 (6.7%)	0 (0%)
Anterior half of tongue	2 (13.3%)	1 (6.7%)
Gingivobuccal region	6 (40.0%)	9 (60.0%)
Lower lip	4 (26.7%)	4 (26.7%)
Lower gingival region	2 (13.3%)	0 (0%)
Right side of tongue	0 (0%)	1 (6.7%)

A higher proportion of patients in the neoadjuvant methotrexate group (33.3%) were managed with WLE alone compared to the primary surgery group (13.3%). Fewer patients in the neoadjuvant group required extensive surgery, such as hemi-mandibulectomy with pectoralis major myocutaneous (PMMC) flap, compared to the primary surgery group (13.3% vs. 33.3%). Other surgical approaches, such as hemi-glossectomy with bilateral SOND and WLE + Right modified radical neck dissection (MRND, were only seen in the neoadjuvant group. The distribution of surgeries suggests a possible trend toward fewer extensive procedures in the neoadjuvant methotrexate group. However, given the small sample size and exploratory nature of this study, this finding should be interpreted with caution and warrants further investigation in larger trials (Table 6).

**Table 6:** Distribution of surgery performed between study groups PMMC - pectoralis major myocutaneous, MRND - Modified Radical Neck Dissection

Surgery performed	Neoadjuvant methotrexate group (N=15)	Surgical management group (N=15)
Wide local excision (WLE)	5 (33.3%)	2 (13.3%)
Wide local excision with SOND	3 (20.0%)	4 (26.7%)
WLE + hemi mandibulectomy + PMMC flap	3 (20.0%)	2 (13.3%)
Hemi-glossectomy with bilateral SOND	1 (6.7%)	0 (0.0%)
Hemi-mandibulectomy + PMMC flap	2 (13.3%)	5 (33.3%)
WLE + Right MRND	1 (6.7%)	0 (0.0%)
Hemi-glossectomy + MRND	0 (0.0%)	2 (13.3%)

In the neoadjuvant methotrexate group, 93.33% of patients had negative free margins after surgery, indicating successful removal of the tumor with clear margins. In contrast, only 53.33% of patients in the primary surgical management group achieved negative free margins, suggesting a higher likelihood of residual tumor presence. The p-value of 0.015 indicates that this difference is statistically significant (Table 7).

**Table 7:** Comparison of negative free margin incidence between study groups P-value was calculated using Fisher’s exact test

Negative free margins	Neoadjuvant methotrexate group (N=15)	Surgical management group (N=15)	P-value
Yes	14 (93.3%)	8 (53.3%)	0.015
No	1 (6.7%)	7 (46.7%)	

Recurrence was observed in 13.3% of patients in the neoadjuvant methotrexate group, indicating a lower incidence of recurrence following treatment compared to the surgical management group, where recurrence was observed in 40% of patients. Although the difference in recurrence rates between the two groups was not statistically significant, with a p-value of 0.104, the trend suggests a potential benefit of neoadjuvant therapy in reducing recurrence (Table 8).

**Table 8:** Comparison of incidence of recurrence between study groups P-value was calculated using Fisher’s exact test

Recurrence	Neoadjuvant methotrexate group (N=15)	Surgical management group (N=15)	P-value
Yes	2 (13.3%)	6 (40.0%)	0.104
No	13 (86.7%)	9 (60.0%)	

Only 13.3% of patients in the neoadjuvant methotrexate group required radiotherapy after surgery, compared to 80% of patients in the surgical management group. This reduction in the need for postoperative radiotherapy indicates a significant advantage of neoadjuvant therapy, with a p-value of 0.0003, demonstrating a statistically significant difference between the two groups (Table 9).

**Table 9:** Comparison of radiotherapy given after surgery between study groups P-value was calculated using the Fisher’s exact test

Received radiotherapy after surgery	Neoadjuvant methotrexate group (N=15)	Surgical Management Group (N=15)	P-value
Yes	2 (13.3%)	12 (80.0%)	0.0003
No	13 (86.7%)	3 (20.0%)	

The study was conducted at Byramjee Jeejeebhoy Government Medical College and Sassoon General Hospital, Pune, where 30 patients diagnosed with stage III oral cavity carcinoma were selected and divided into two groups, each comprising 15 patients. The groups were compared to evaluate the effectiveness of neoadjuvant methotrexate therapy followed by surgery versus primary surgical management. Our findings revealed that patients who received neoadjuvant chemotherapy exhibited improved oncological outcomes, with significant downstaging of the disease and reduced recurrence rates. Furthermore, patients in the neoadjuvant group required less extensive surgical procedures in comparison to those in the primary surgery group.

In the neoadjuvant group, 33.3% (n=5) of patients were able to undergo WLE due to tumor downstaging, in contrast to 13.3% (n=2) in the surgical group. This reduction in the need for more invasive surgeries resulted in shorter postoperative stays and fewer complications. Extensive procedures such as hemi-mandibulectomy with PMMC flap reconstruction, commonly performed in the surgical group, were less frequently required in the neoadjuvant group. Additionally, 93.33% (n=14) of patients in the neoadjuvant group achieved negative surgical margins, compared to 53.33% (n=8) in the surgical group, indicating better surgical outcomes for the patients receiving neoadjuvant therapy.

The study also demonstrated a significant reduction in the need for postoperative radiotherapy in the neoadjuvant group, where only 13.3% (n=1) of patients required radiotherapy, compared to 53.33% (n=8) in the surgical group. This reduction improved oncological outcomes and reduced patient morbidity, contributing to better cost-efficiency in treatment. Another important observation was that two cycles of methotrexate were sufficient to downstage the disease and achieve better surgical and oncological outcomes, in contrast to the standard four-cycle regimen of cisplatin and 5-fluorouracil [19].

## Discussion

The results of this study demonstrate the effectiveness of neoadjuvant methotrexate therapy in patients with stage III oral carcinoma. Our findings align with recent research showing that neoadjuvant chemotherapy can significantly downstage tumors, thereby facilitating less extensive surgical procedures and improving overall oncological outcomes. In particular, the significant reduction in the need for hemi-mandibulectomy and PMMC flap reconstruction in the neoadjuvant group compared to the primary surgery group indicates the clinical utility of methotrexate in reducing tumor burden preoperatively [20].

The high percentage of negative surgical margins in the neoadjuvant group (93.33%) versus the surgical group (53.33%) suggests that methotrexate improves the precision of surgical excision, leading to better outcomes and reduced recurrence rates. This observation echoes the findings of recent studies, which highlight the importance of achieving negative margins in improving long-term survival and reducing postoperative morbidity [21].

Additionally, the reduced need for postoperative radiotherapy in the neoadjuvant group (13.3%) compared to the surgical group (53.33%) is another significant advantage. This outcome is consistent with literature that demonstrates a reduced reliance on adjuvant therapies when effective neoadjuvant chemotherapy is employed [22]. While the reduction in locoregional recurrence was not statistically significant in this study, the trend suggests that methotrexate could play a valuable role in reducing recurrence in the long term [23].

Limitations

Despite the positive findings, several limitations must be acknowledged. The small sample size of 30 patients limits the statistical power to detect subtle but meaningful differences, increasing the risk of type II errors. This study is exploratory and intended to generate preliminary insights rather than provide definitive conclusions, and larger trials are needed to confirm these results.

The study’s single-center design restricts the generalizability of the findings, as outcomes may differ based on patient demographics, surgical techniques, and healthcare resources in other settings. Although methotrexate was chosen due to patient-specific factors like comorbidities, it is not the standard first-line neoadjuvant therapy for oral carcinoma, with cisplatin-based regimens being preferred. This may introduce bias, as methotrexate does not reflect current clinical guidelines.

There were also variations in TNM staging and tumor location between groups, which could have influenced the outcomes despite randomization. The observed reduction in postoperative radiotherapy use in the methotrexate group, while statistically significant, may reflect individual patient factors and surgical outcomes rather than direct treatment efficacy. Additionally, surgical variability may have affected margin status and recurrence rates, which are known predictors of treatment success.

Some results, such as recurrence rates and radiotherapy usage, showed non-significant or borderline p-values, suggesting these findings are hypothesis-generating rather than conclusive. Finally, as the study was conducted between 2018 and 2020, evolving guidelines and newer chemotherapy protocols may limit the relevance of methotrexate today. A longer follow-up period is also needed to assess the therapy’s sustained impact on recurrence and survival. These limitations highlight the need for larger, multi-center trials to validate the findings and optimize treatment protocols.

## Conclusions

The management of stage III oral cancer involves multiple treatment modalities, with ongoing research focused on optimizing outcomes through surgery, chemotherapy, and radiotherapy. Our study showed significant improvement in oncological and surgical outcomes for patients getting neoadjuvant chemotherapy with two cycles of methotrexate followed by surgical resection. These patients had effective downstaging of tumors, thereby requiring less morbid procedures like WLE for tumor resection. The neoadjuvant methotrexate group demonstrated trends toward improved surgical outcomes with a higher proportion of negative margins and fewer locoregional recurrences. However, given the small sample size and limited statistical power, these findings require validation through larger studies.

Furthermore, this form of treatment showed reduced locoregional recurrences. An added finding was the efficacy of using methotrexate as a neoadjuvant chemotherapy treatment for stage III oral carcinoma, offering fewer side effects, and better surgical and oncological outcomes. Methotrexate shows promise in facilitating surgical resection for select patients with stage III oral carcinoma who are not suitable for cisplatin-based chemotherapy. Further research and randomized controlled trials are required to confirm this.
